# A proven reoffending study of individuals managed under the multi-agency public protection arrangements (MAPPA) in England and Wales

**DOI:** 10.3389/fpsyg.2024.1371023

**Published:** 2024-04-10

**Authors:** Samantha Lundrigan, Natalie Mann, David Specht, Lea C. Kamitz

**Affiliations:** Policing Institute for the Eastern Region, Anglia Ruskin University, Chelmsford, United Kingdom

**Keywords:** MAPPA, sex offenders, violent offenders, recidivism, proven reoffending, crime harm

## Abstract

**Introduction:**

Past research into the effectiveness of multi-agency public protection arrangements (MAPPA) in reducing reoffending it limited. Thus, the current study aimed to evaluate proven reoffending patterns for MAPPA managed individuals.

**Methods:**

Proven reoffending for 39,501 MAPPA managed individuals was investigated by (1) examining patterns in the timing and frequency of proven reoffending for MAPPA managed individuals; (2) examining 1-, 3-, and 5-year proven reoffending patterns of MAPPA managed individuals by MAPPA category, age, and gender; and (3) comparing crime harm levels and recall to custody for MAPPA managed individuals pre- and post-MAPPA adoption.

**Results:**

Taken together, our findings show that proven reoffending rates for individuals managed under MAPPA are substantially lower than those reported in proven reoffending statistics for England and Wales.

**Discussion:**

Our results suggest that MAPPA is making a positive contribution to a managing individuals convicted of sexual and violent offenses. Additionally, our findings provide the best evidence to date that MAPPA management may also be effective at reducing less serious offenses which do not typically involve immediate removal from society. These findings are considered in light of their theoretical and practical implications while potential limitations and avenues for future research are outlined.

## Introduction

Multi-agency public protection arrangements (MAPPA) were introduced in England and Wales in 2003 under the Criminal Justice Act (2003, p. 325–327), with the aim of strengthening the monitoring of community-based individuals convicted of sexual and violent offenses. Under MAPPA, the Police, the Probation Service and the Prison Service, along with other agencies who have a “duty to cooperate”^1^ (DTC hereafter), are legally required to work together to monitor and manage the risk posed by such individuals (Ministry of Justice, 2021). There are three categories of MAPPA management: Category 1: registered sexual offenders; Category 2: violent offenders sentenced to 12 months or more in custody (immediate or suspended) or detained under a hospital order, and Category 3: other dangerous offenders who pose a serious risk of harm.^2^ Under MAPPA, individuals are managed at one of three levels, which reflects the level of multi-agency cooperation required to implement their risk management plan effectively. Individuals may move between the levels, reflecting changes in the level of risk they are deemed to present. Level 1 provides multi-agency support for lead agency risk management with information sharing; level 2 provides formal multi-agency meetings, including active involvement of more than one agency to manage the individual; and level 3 provides formal multi-agency meetings and extra resources, the “Critical Few” including Critical Public Protection Cases (CPPC) (Ministry of Justice, 2021).

MAPPA is responsible for protecting society from some 66,741 individuals convicted of sexual offenses and some 22,697 individuals convicted of violent offenses or considered dangerous (Ministry of Justice, 2022a). The effectiveness of MAPPA in preventing reoffending has been the subject of much scrutiny in recent years, after a series of high-profile crimes committed by offenders on release into the community and under MAPPA supervision. For example, inquests into the deaths of Saskia Jones and Jack Merritt in the Fishmongers' Hall terror attack found that there were serious deficiencies in the management of the perpetrator by MAPPA (HM Coroner, 2021). As a result, a number of reviews of MAPPA have been undertaken including the Terrorist Risk Offenders: Independent Review of Statutory Multi-Agency Public Protection Arrangements (Hall, 2020), HMPPS Review of MAPPA Level 1, the HM Joint Thematic Inspection of Police and the Probation Service (HM Inspectorate of Probation, 2022), and the Independent Review into the Police-led Management of Registered Sex Offenders in the Community (Creedon, 2022). Despite this scrutiny however, little is currently known about how effective MAPPA is at reducing re-offending.

### Prior research on the effectiveness of MAPPA in reducing reoffending

Past research into the effectiveness of MAPPA on reducing reoffending is limited to two Ministry of Justice studies (Peck, 2011; Bryant et al., 2015). Peck (2011) examined reconviction patterns of MAPPA managed individuals from 2001 to 2004 and compared them to a different cohort of pre- MAPPA managed individuals from 1998 to 2000. They found that those released from custody between 2001 and 2004 (i.e., after the implementation of MAPPA) had a lower 1-year reconviction rate than those released between 1998 and 2000 and this remained true at the 2-year follow-up. The general reconviction rate of the 1998 cohort was 26.4%, but for the 2004 cohort it had reduced to 19.9%, a 6.5% point reduction.

In an update to Peck's (2011) study, Bryant et al. (2015) examined 1-year proven reoffending rates for new MAPPA Category 1 and 2 eligible individuals from 2000 to 2010 and compared differences in reoffending rates for MAPPA eligible individuals and a comparison group to estimate the impact of MAPPA on reducing reoffending. They found a 3% decrease in the 1-year proven reoffending rates for Category 1 and Category 2 individuals between 2000 and 2004. The authors concluded that, between 2000 and 2010, MAPPA may be associated with a four-percentage point reduction in proven reoffending for new MAPPA eligible individuals, and a two-percentage point reduction in serious reoffending. Table 1 summarizes the two studies.

**Table 1:** Summary of previous MAPPA reoffending research.

	**Peck (2011)**	**Bryant et al. (2015)**
Research design	MAPPA subject group vs. non-MAPPA comparison group	MAPPA subject group vs. non-MAPPA comparison group
Sample selection	MAPPA eligible	New MAPPA eligible offenders
MAPPA categories	Cat 1, Cat 2, Cat 3	Cat 1, Cat 2
Total sample size	67, 679	136, 000
Time frame	1998–2004	2000–2010
Reoffending measure(s)	1- and 2-year reconviction rates	1-year proven reoffending rates
Exclusions	Released from hospitals, registered sexual offenders released before 1998 or given community sentences, offenders given life sentences, certain Category 3 offenders	Category 3 offenders
Results	Offenders released from custody between 2001 and 2004 (i.e., after the implementation of MAPPA management) had a lower 1-year reconviction rate than those released between 1998 and 2000. Remained true at the 2-year follow-up. The general reconviction rate of the 1998 cohort was 26.4%, but for the 2004 cohort it had reduced to 19.9%, a 6.5% point reduction.	The 1 year proven reoffending rate amongst Category 1 offenders decreased from 13% in 2000 to 10% in 2004. Gradually increased back to 13% in 2010. The 1-year proven reoffending rate amongst Category 2 offenders decreased from 26% in 2000 (pre-implementation of MAPPA management) to 23% in 2004. Fluctuated between 22% and 24% from 2004 to 2010. MAPPA associated with a 4% reduction in proven reoffending by new MAPPA eligible offenders, and a 2% reduction in serious reoffending, from 2000 to 2010.

Whilst the two studies discussed have gone some way to help understand the impact of MAPPA management on reoffending, there are several limitations to both. The first relates to the reoffending measures used. Peck (2011) calculated reconviction rates which included convictions but not cautions. Whilst the more recent study by Bryant et al. (2015) utilized proven reoffending rates which provide a more complete picture of reoffending, both studies predominantly examined 1-year rates which, given that individuals convicted of sexual offenses do not tend to reoffend for several years (Soothill et al., 2000; Cann et al., 2004), may be problematic. The second relates to study samples. Neither study included all types of individual subject to MAPPA management. Peck (2011) included only those individuals who had served a period of incarceration, excluding all those serving community sentences or hospital orders. Bryant et al. (2015) included only Category 1 and Category 2 managed individuals, excluding all those in Category 3. Additionally, the sampling time frame used by Bryant et al. (2015) covered an unstable period for MAPPA during which guidance on MAPPA eligible offenses was still evolving. As such, some individuals would have been managed very differently to others depending on the guidance at the time. Both studies are also now outdated. Peck (2011) utilized data from 2000 to 2004, and Bryant et al. (2015) utilized data from 2000 to 2010, leaving a further 11 years of data unexamined.

### The current study

Approaching 20 years since the inception of MAPPA, the Responsible Authority National Steering Group (RANSG hereafter) at the Ministry of Justice requested a comprehensive examination of MAPPA effectiveness which would address the shortcomings of previous research, and which could be utilized to inform its future structure and operation. Thus, the National MAPPA Research was undertaken between January 2020 and November 2022. The research was made up of three complimentary but overlapping components: a proven reoffending analysis of over 70,000 MAPPA managed individuals (the current paper); a process effectiveness analysis (Mann and Lundrigan, 2023) and a MAPPA serious case review (SCR hereafter) analysis (Mann et al., 2023).

The aim of the current research was to examine proven reoffending patterns for MAPPA managed individuals to evaluate the effectiveness of MAPPA in reducing reoffending. To address this aim, we:

- Examined patterns in the timing and frequency of proven reoffending for MAPPA managed individuals;
- Examined 1-, 3- and 5-year proven reoffending patterns of MAPPA managed individuals by MAPPA category, age, and gender; and
- Compared crime harm levels and recall to custody for MAPPA managed individuals pre- and post-MAPPA adoption.

## Method

### Ethics

Approval from both Anglia Ruskin University's Faculty Research Ethics Panel and HMPPS' Research Ethics Committee (2020-102) was secured. All ethical issues pertinent to this study, such as anonymity, data security and management were considered and managed accordingly. A Data Sharing Agreement was put in place between ARU and the National Police Chief's Council (June 2021). This agreement covered all data processing and data management arrangements for the duration of the project.

### Dataset

For the purposes of this research, it was necessary to create a bespoke dataset combining data from three existing administrative data sources: ViSOR,^3^ The Police National Computer (PNC)^4^ and the Probation Service's database n-Delius.^5^ The following steps were undertaken to arrive at the final sample ready for analysis:

(1) A ViSOR data extraction exercise was undertaken by the national ViSOR team on November 6, 2021. The data extraction included all individuals subject to MAPPA management for the first time between 2003 (the beginning of MAPPA) and 2014^6^ with reoffending data up to the date of extraction (*N* = 139,978).^7^ When the research was approved in 2019, we aimed to examine 5-year proven reoffending using the most up-to-date data available. As such, we applied for data on all individuals who were new to MAPPA management up to 2014 and proven reoffending data up to the data extraction date (2019).
(2) Individuals with incomplete data regarding their MAPPA category were removed, leaving only individuals with valid MAPPA categories (*n* = 81,705).
(3) Those individuals who were new to MAPPA, or who had their MAPPA status reactivated, between January 1, 2012 and December 31, 2014 were identified (*n* = 31,541).
(4) The sample identified from ViSOR were cross referenced with n-Delius to identify any additional Category 2 managed individuals not recorded on ViSOR.^8^ This added an additional 7,960 new individuals with valid MAPPA levels (*n* = 39,501).
(5) The sample was then cross referenced with PNC data to ensure a complete record of all offending (both prior to and after the date of MAPPA registration), rather than the limit of the first 50 convictions which is held on ViSOR.
(6) The last step involved identifying MAPPA individuals who had undertaken the now-discredited (Mews et al., 2017) Sex Offender Treatment Programmes (SOTP and iSOTP). Data on programme completions was provided by the Reducing Reoffending and Probation Data and Statistics Team. The PNC IDs of 11,667 Category 1 (Registered Sex Offenders) and 2 (individuals convicted of sexual offenses who did not meet notification requirements) managed individuals were provided to the Data Science and Personalisation Hub at the Ministry of Justice who returned the IDs of those who had completed the programme (*n* = 798). Analysis carried out including and excluding this group of individuals showed no significant differences in findings and so this group were included in the final sample.

### Participants

The final sample included 39,501 individuals with a MAPPA adoption date between January 1, 2012 and December 31, 2014. It included those individuals subject to MAPPA management living abroad and managed by ACRO criminal records service. Table 2 shows the breakdown of the sample by MAPPA category and level.

**Table 2:** Sample frequencies for MAPPA categories and levels.

**MAPPA category**	**MAPPA level**				**Total**
	**Level 1**	**Level 2**	**Level 3**	**Not known**	
Category 1	25.72% (10,160)	1.09% (430)	0.07% (24)	30.02% (11,858)	56.89% (22,472)
Category 2	22.98% (9,079)	6.71% (2,650)	0.27% (105)	9.77% (3,858)	39.73% (15,692)
Category 3	0.07% (26)	1.44% (569)	0.07% (24)	1.87% (738)	3.44% (1,357)
Total	48.77% (19,265)	9.35% (3,694)	0.39% (153)	41.65% (16,454)	100% (39,501)

Represent the percentage of individuals from the study sample in each level or category. Numbers of individuals in each level or category are in parentheses.

Of the whole sample, 99.7% remained under MAPPA management at the point of data extraction, with only 99 individuals no longer being managed under MAPPA, showing that most individuals had been managed under MAPPA for between 7 and 9 years (*M* = 8.08, *SD* = 0.87). Of the whole sample, 26.7% had served one or more custodial sentences during their MAPPA management (up to date of data extraction). Table 3 depicts the demographic characteristics of the sample. As can be seen, there was missing data for several of the demographic variables.

**Table 3:** Sample demographics.

**Demographic factor**	**Categories**	**% (*n*)**
Age	Juvenile	2.82 (1,113)
	Adult	94.45 (37,310)
	Missing	2.58 (1,078)
Gender	Male	95.73 (37,814)
	Female	3.69 (1,456)
	Other	0.51 (200)
	Missing	0.08 (31)
Ethnicity	White	68.07 (26,888)
	Black	7.56 (2,986)
	Asian	5.21 (2,058)
	Mixed ethnicity	1.50 (593)
	Missing	17.71 (6,994)
Marital status	Single	16.04 (6,335)
	Married /civil partner/ lives with partner	7.81 (3,084)
	Divorced/ separated/ estranged	5.34 (2,110)
	Widowed	0.45 (185)
	Missing	70.35 (27,787)
Education level	None	8.50 (3,356)
	GCSE	4.08 (1,613)
	BTech	0.03 (10)
	Diploma	0.78 (308)
	A level	0.86 (339)
	Degree	1.19 (469)
	PhD	0.07 (29)
	Missing	84.50 (33,377)
Mental health	Depression	61.32 (1,341)
	Personality Disorder	6.72 (147)
	Schizophrenia	6.90 (151)
	Psychosis	1.87 (41)
	Paranoia	1.78 (39)
	Other	42.80 (936)
Drug use	Cannabis	68.88 (1,268)
	Cocaine	21.40 (394)
	Ketamine	1.41 (26)
	Amphetamines	9.29 (171)
	Heroin	11.95 (220)
	Ecstasy	6.68 (123)
	Magic Mushrooms/LSD	1.09 (20)
	Methadone	2.77 (51)
	Other	33.08 (609)
Veteran	Past or current military service	0.57% (227)

Percentages are calculated from totals where data recorded is not the total sample.

### Analysis

To examine patterns in reoffending for MAPPA-managed individuals, as well as the potential effect MAPPA might have on such reoffending, this research used four different measures: Proven reoffending rates, offending intensity, recall to custody, and the Crime Harm Index.

#### Proven reoffending rates

For the purposes of this study, a proven reoffence was defined as any offense receiving a court conviction or a police caution after an individual's date of MAPPA registration. For every individual in the sample, we had proven reoffending data from their date of adoption under MAPPA (January 1, 2012–December 31, 2014) until the data extraction date (November 6, 2021).

Proven reoffending was calculated in several stages. Firstly, PNC IDs and MAPPA adoption dates were loaded. This allowed a mapping between the individual subject to MAPPA management and any recorded offenses from either the PNC or ViSOR databases. For each PNC ID, all offense occurrence dates were inputted for that individual. The proven reoffending for a particular individual was then calculated by searching for any convictions or cautions which occurred within *N* years after the individual's MAPPA adoption date, with *N* in the range of 1 to 5 years, in 6-month intervals. Any prison sentences during the reoffending timeframe were taken into consideration by increasing the allotted timeframe over which the individual could commit an offense; hence the reoffending timeframe was taken to be *N* years of community management time.^9^

A complimentary measure of differences in reoffending rates between groups was also carried out using the Cox Proportional Hazards model. Although we could not compare the hazard rates for reoffending between MAPPA-managed and non-MAPPA-managed due to the absence of a control sample, the proportional hazard rates were nonetheless evaluated for comparison to the general reoffending trends.

#### Offending intensity

The first offending intensity measure was the number of offenses committed by an individual post MAPPA registration. A second measure of offending intensity involved calculating the average number of reoffences committed post MAPPA registration.

#### Recall to custody

The recall to custody measure is the percentage of individuals who were recalled to custody,^10^ at least once, whilst under MAPPA management.

#### Crime Harm Index

The “Crime Harm Index” or “Cambridge CHI” (Sherman et al., 2016) provides a framework for the comparative analysis of the harm that different crimes inflict. The Cambridge CHI acknowledges that crimes pose different levels of harm and provides a weighted index to measure how harmful different crimes are in proportion to the others. This approach adds a larger weight to more harmful crimes (e.g., homicide, rape, and grievous bodily harm with intent), distinguishing them from less harmful types of crime (e.g., minor thefts, criminal damage, and common assault). The Cambridge CHI is calculated by multiplying each crime event in each crime category by the number of days in prison that it would attract for a convicted perpetrator, using sentence starting points as the baseline penalty for each offense (Sherman et al., 2016). For offenses where the starting point is either a community sentence or a fine, scores based on the number of hours required to complete the work component of a community sentence or to work to pay off the fine are used. For example, the CHI value is 10 for theft of a vehicle, 365 for robbery, and 2190 for rape of a child under the age of 13. For the crime harm analysis in the current study, the same data retrieval process was employed as in the proven reoffending analysis. The Cambridge CHI (2020) was used to map criminal justice codes to crime harm for each MAPPA managed individual who reoffended.

## Results

This section begins by presenting results on the timing and frequency of proven reoffending for MAPPA managed individuals. This is followed by the analysis of proven reoffending patterns by MAPPA category, gender, and age. Finally, the results of the pre- and post-MAPPA recall to custody and crime harm analysis are presented. Examination of the entire sample from adoption date to data collection date revealed that 31.1% (*n* = 12,357) of the whole sample had at least one proven reoffence over the period under consideration.

### Timing and frequency of proven reoffending

#### Time until first proven reoffence

**Figure 1:**
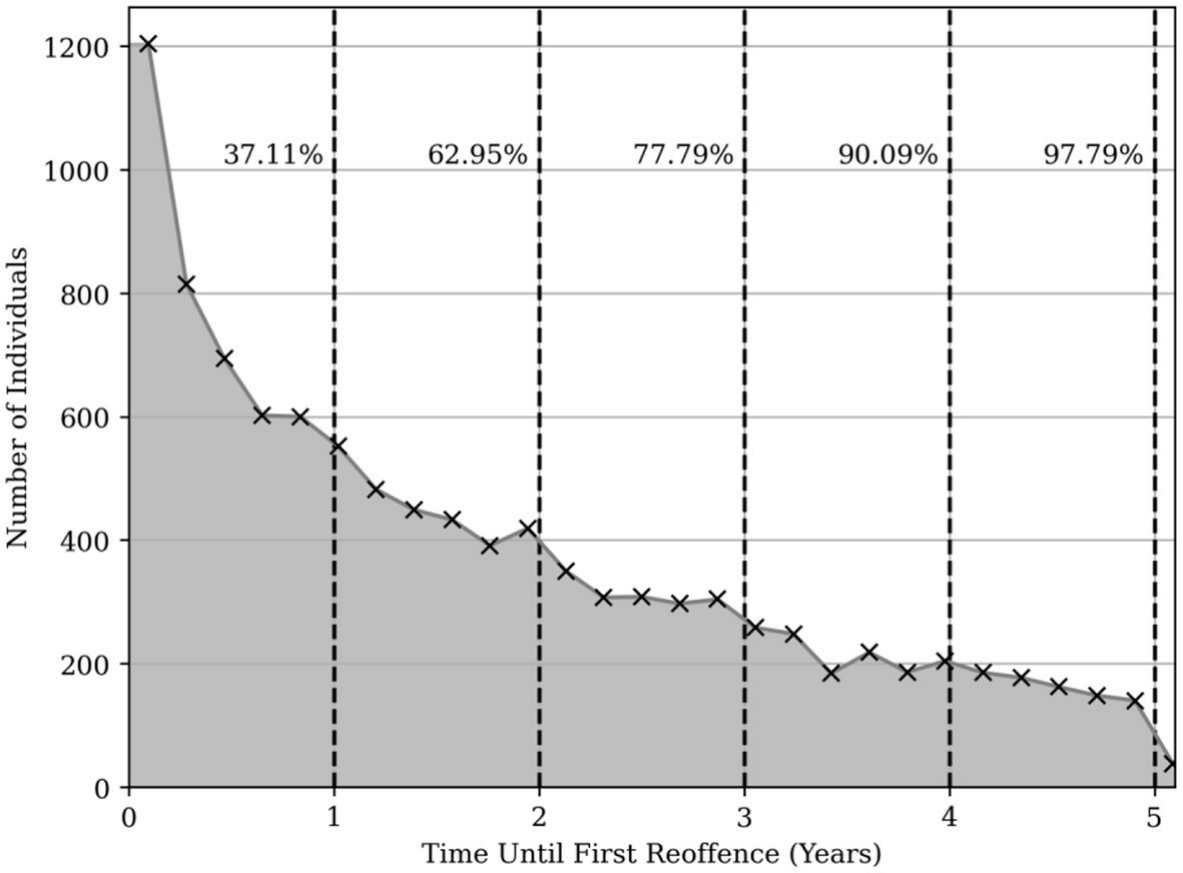
Time to first reoffence for MAPPA managed individuals who have reoffended. Black dashed lines are shown at each year to indicate the cumulative sum of individuals who had reoffended up to that point.

Figure 1 illustrates the distribution of time lapsed between MAPPA adoption date and the first reoffence.

At the end of the first year of registration, 37.1% of those MAPPA managed individuals who re-offended within 5 years had committed their first reoffence. This rose to 63.0% at the end of year two, and so on reaching 97.8% at the end of year five.

**Figure 2:**
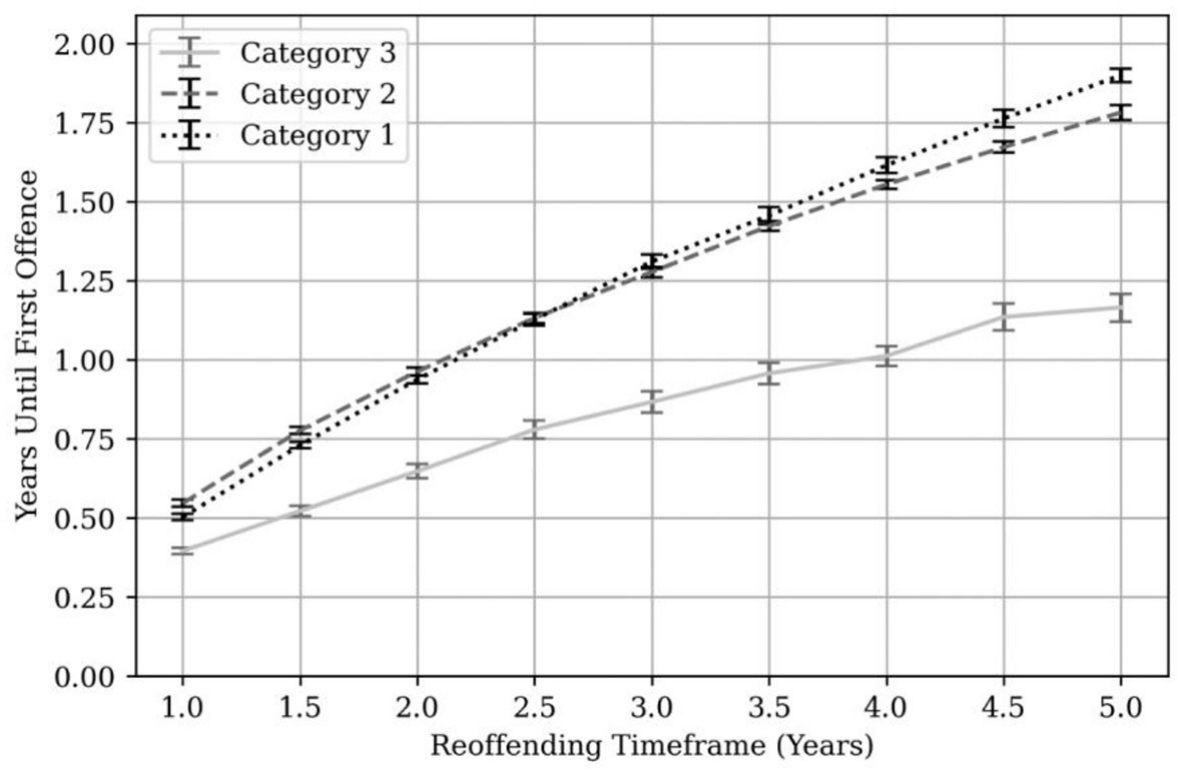
Average time to first reoffence by MAPPA category. Error bars are shown in black and gray at each 6-month time point.

Figure 2 shows the average time lapse between MAPPA adoption date and first re-offense broken down by MAPPA category.

At 1-year post-MAPPA, the average time to first reoffence was 0.48 years for Category 1 managed individuals, 0.53 years for Category 2 managed individuals and 0.36 years for Category 3 managed individuals. At 3 years post-MAPPA, the average time to first re-offense was 1.26 years for Category 1, 1.26 years for Category 2 and 0.82 years for Category 3 managed individuals. At 5 years, this rose to 1.82 years for Category 1, 1.76 years for Category 2 and 1.10 years for Category 3 managed individuals.

#### Proven reoffending frequency

**Figure 3:**
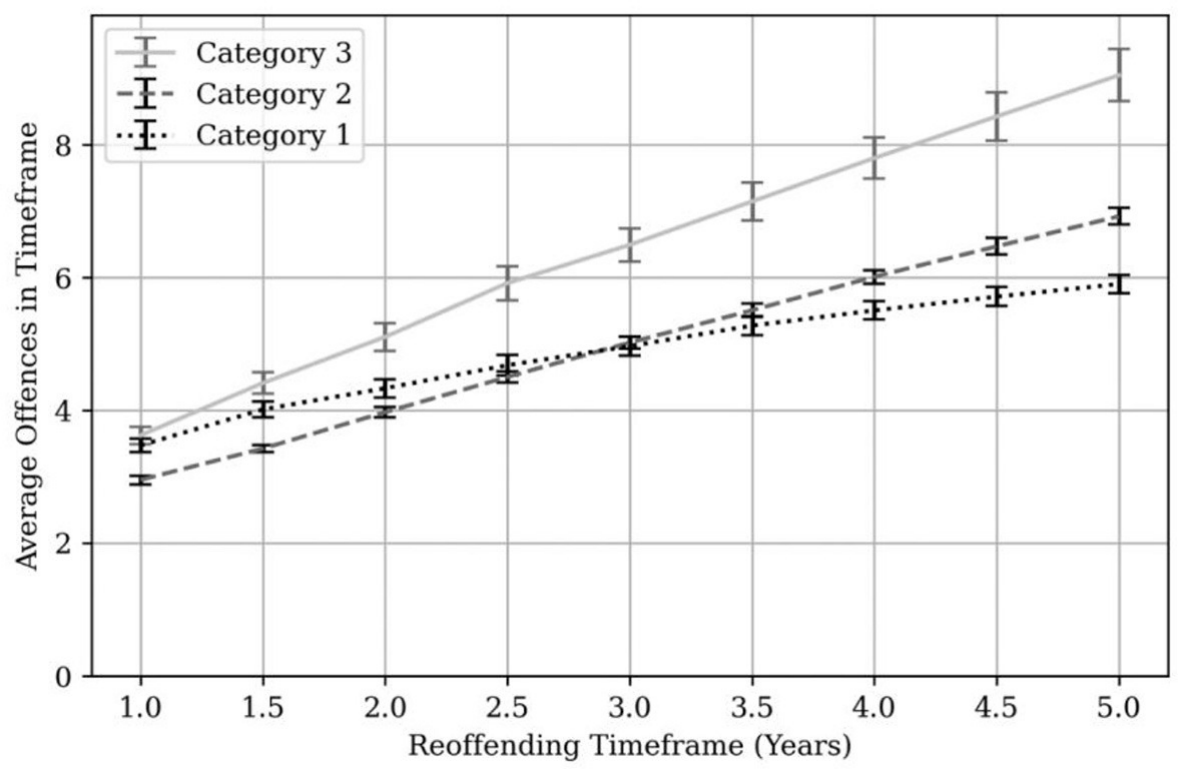
Proven reoffending frequency by MAPPA category. Error bars are shown in black and gray at each 6-month time point.

Figure 3 shows the average number of proven reoffences broken down by MAPPA category.

Across all those MAPPA managed individuals who reoffended, the average number of proven reoffences was 3.62 offenses at year one, 5.59 offenses at year three and 7.12 offenses at year five. At one year, Category 1 managed individuals committed an average of 3.78 offenses each; Category 2 committed an average of 3.14 offenses each and Category 3 committed an average of 4.19 offenses each. At 3 years this rose to 5.40 (Category 1), 5.42 (Category 2) and 7.43 (Category 3) offenses. At 5 years this rose again to 6.40 (Category 1), 7.50 (Category 2) and 10.26 (Category 3) offenses each. For comparison, the average number of reoffences per reoffending individual in England and Wales ranged from 3.30 to 3.78 reoffences per year over a similar period (Ministry of Justice, 2022b).^11^

### Proven reoffending rates

#### Overall proven reoffending

**Figure 4:**
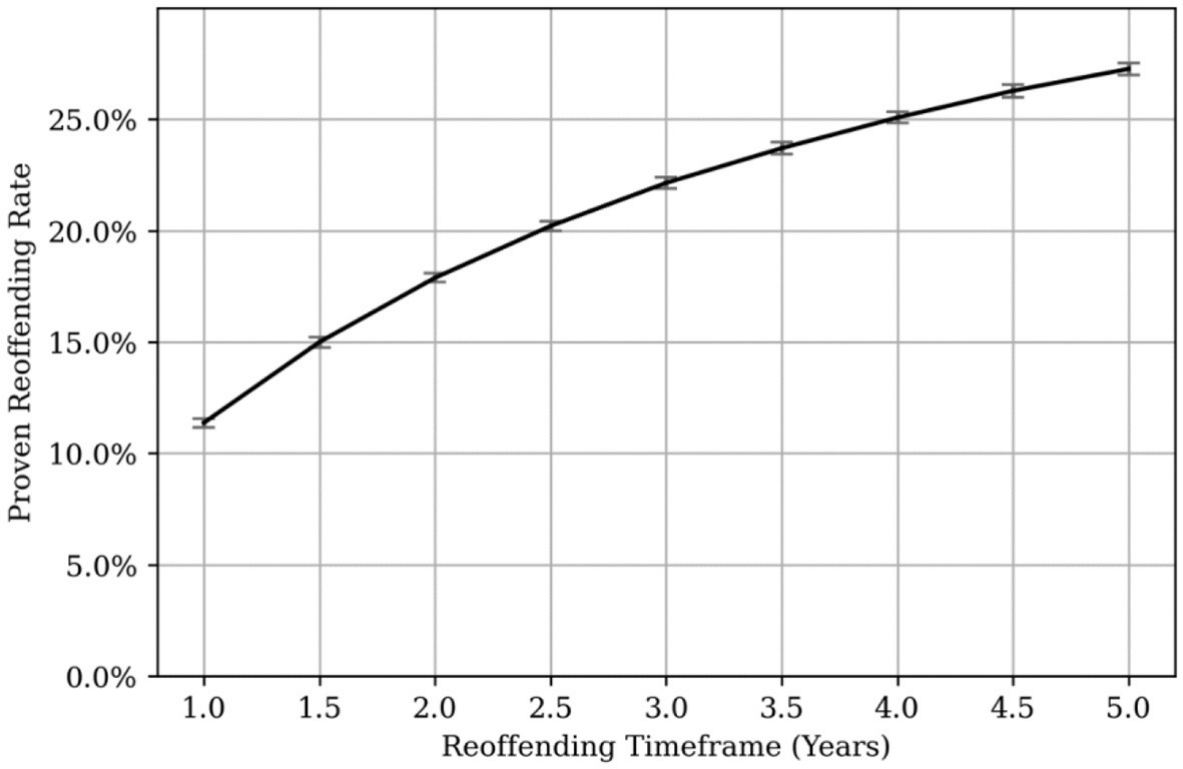
Proven reoffending rates for MAPPA sample. Error bars are shown in red at each 6-month time point.

The proven reoffending rates for the whole sample across the 5-year period are shown in Figure 4.

At 1-year post-MAPPA adoption the proven reoffending rate was 12.3%, and at 3 years 23.0%, and 5 years 28.1%. For comparison, the annual average, 1-year “general offender population” proven reoffending rate over a similar period ranged from 30.0% to 31.3% (Ministry of Justice, 2022b). The MAPPA 1-year proven reoffending rate is also significantly lower than that found by Bryant et al. (2015), which ranged from 25 to 30% for MAPPA managed individuals between 2000 and 2010, and lower than the reconviction rate of 19.9% found by Peck (2011) for a 2004 MAPPA managed cohort.

#### Proven reoffending by MAPPA category

**Figure 5:**
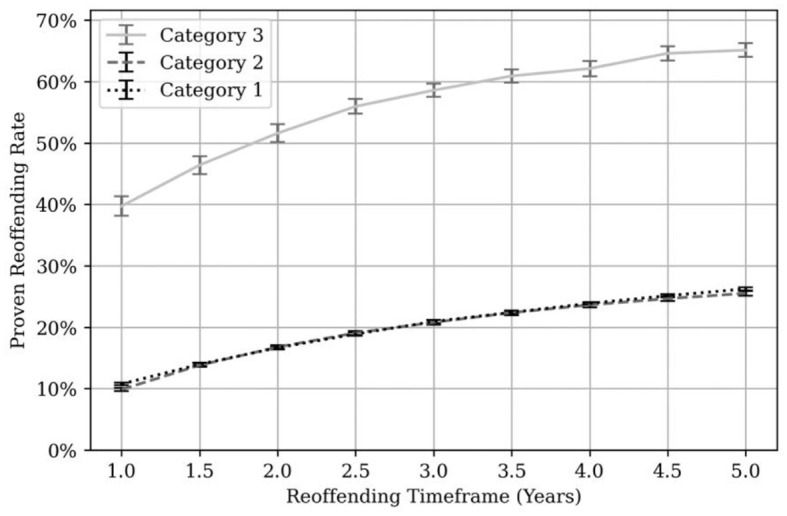
Proven reoffending rates by MAPPA category. Error bars are shown in black and gray at each 6-month time point.

Figure 5 shows proven reoffending rates by MAPPA category.

At each time point, proven reoffending rates are highest for Category 3 managed individuals ranging from 41.9% at 1 year to 60.1% at 3 years and 66.2% at 5 years. Proven reoffending rates for Category 1 and Category 2 managed individuals are significantly lower. For Category 1, the 1 year proven reoffending rate was 11.9%, the 3-year rate was 22.1% and the 5-year rate was 27.3%. For Category 2, the 1 year proven reoffending rate was 10.2%, the 3-year rate was 21.1% and the 5-year rate was 25.8%.

By comparison, Bryant et al. (2015) found proven reoffending rates between 2000 and 2010 fluctuated between 10% and 15% for Category 1 managed individuals and 22%−26% for Category 2 managed individuals. By way of further comparison, the annual average, 1-year proven reoffending rate nationally for individuals convicted of a sexual index offense ranged from 13.7% to 15.0% over a similar time-period (Ministry of Justice, 2022b). The same statistics for individuals convicted of violence against the person index offense ranged from 23.5% to 24.7% (Ministry of Justice, 2022b), lower than those found here.

#### Proven reoffending by gender

**Figure 6:**
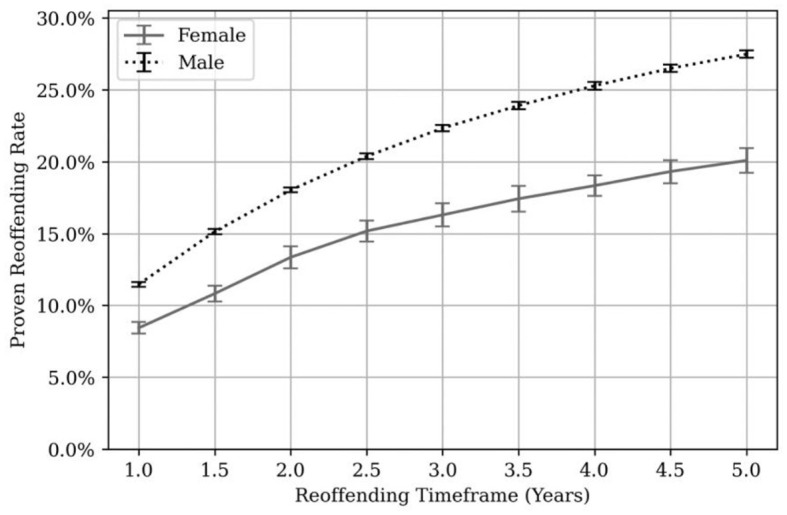
Proven reoffending rates by gender. Error bars are shown in black and gray at each 6-month time point.

Figure 6 shows proven reoffending rates broken down by gender.

At each time point, proven reoffending rates were significantly higher for males than females. The 1-year proven reoffending rate was 12.4% for males and 9.1% for females. The 3-year and 5-year proven reoffending rates were 23.2% and 28.3% for males and 17.2% and 20.7% for females, respectively. It was not possible to calculate reoffending rates for individuals identifying as any other gender due to small numbers.

Bryant et al. (2015) reported proven reoffending rates of between 27 and 30% for males and 22%−28% for females. The 1-year, “general offender population” proven reoffending rates for a similar time-period ranged from 30.3% to 31.8% for males and 21.8% to 22.9% for females (Ministry of Justice, 2022b).

#### Proven reoffending by age

**Figure 7:**
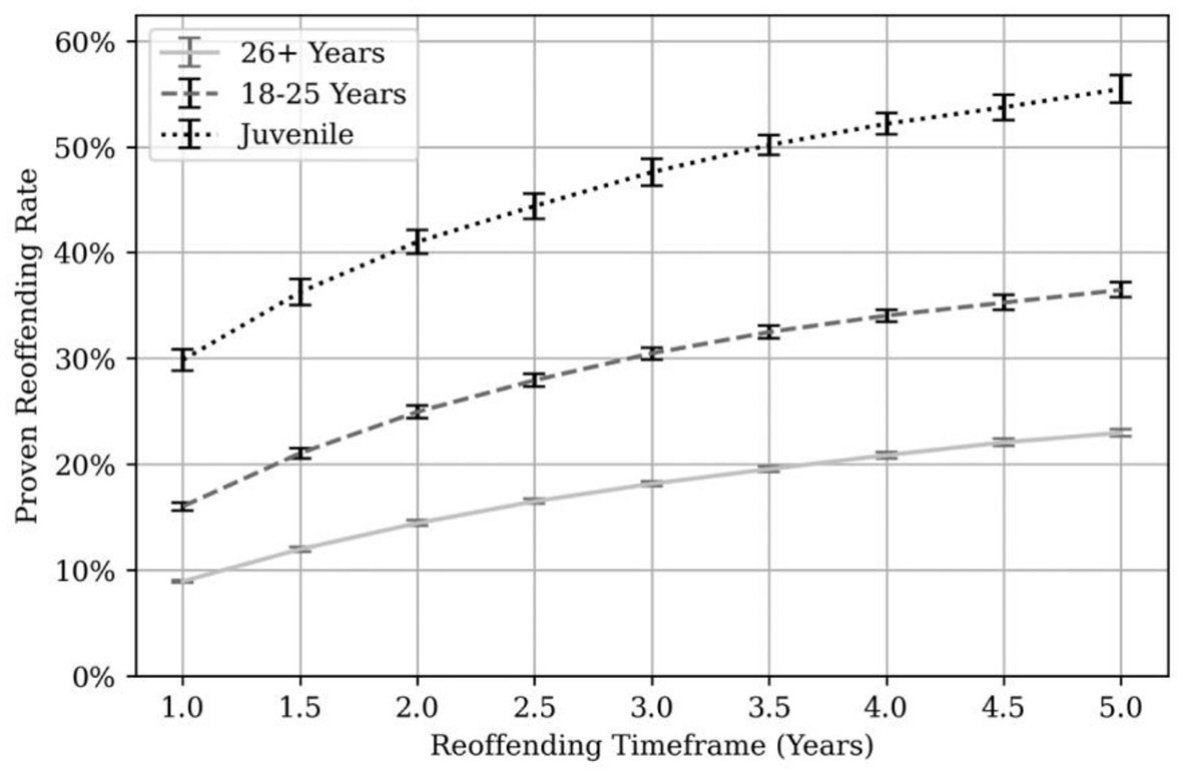
Proven reoffending rates by age at MAPPA adoption. Juveniles = individuals aged 17 or younger at time of adoption under MAPPA. Error bars are shown in black at each 6-month time point.

Figure 7 shows proven reoffending rates by age at adoption under MAPPA. Individuals were split into three age groups, by their age at adoption under MAPPA: 17 years and younger, 18–25 years and 26 years and older.

Proven reoffending rates were significantly higher at each time point for MAPPA managed individuals aged 17 and under with a proven reoffending rate of 33.2% at 1 year, 49.3% at 3 years and 56.3% at 5 years. The lowest proven reoffending rates were found in the 26 + age group (9.6% 1 year, 18.9% 3 years, 23.7% 5 years). For the 18 to 25 age group, the proven reoffending rates were 17.2% at 1 year, 31.6% at 3 years, and 37.4% at 5 years. In comparison, the 1-year, “general offender population” proven reoffending rates for a similar period ranged from 28.9% to 30.0% for adults and 40.4% to 42.9% for juveniles (Ministry of Justice, 2022b).

#### Cox proportional hazards analysis

For a complex system such as the cohort we are analyzing, the assumption of proportional hazards may not hold for each variable analyzed. For example, Figure 6 suggests that reoffending rates of Category 1 managed individuals are initially higher than that of Category 2, but when given a 5-year reoffending timeframe, this ratio is reversed, with Category 2 managed individuals exceeding Category 1. With that in mind, the hazard rates reported in Table 4 largely agree with the reoffending trends reported. For example, comparing Category 1 with Category 3, the hazard rate (*e*^β^) is 1.2 times higher on average for Category 3 (1.79/1.48).

**Table 4:** Cox proportional hazards analysis of the MAPPA sample.

	**β**	**β 95% CI**	**e^β^**	**e^β^ 95% CI**	***p***
Category 1	0.39	0.21, 0.58	1.48	1.23, 1.78	< 0.001
Category 2	−0.45	−0.63, −0.27	0.64	0.53, 0.77	< 0.001
Category 3	0.58	0.37, 0.79	1.79	1.45, 2.21	< 0.001
Male	0.16	−0.30, 0.62	1.17	0.74, 1.86	0.50
Female	−0.16	−0.62, 0.30	0.85	0.54, 1.35	0.50
Age	−0.03	−0.033, −0.028	0.97	0.968, 0.972	< 0.001
Level	1.02	0.96, 1.08	2.77	2.61, 2.93	< 0.001

### Recall to custody

The average recall to custody rate per year for the sample was 0.18% pre-MAPPA and 1.74% post-MAPPA. The ratio of pre- to post-MAPPA per year recall rates was 9.67 demonstrating that post-MAPPA recall rates were nearly 10 times higher than pre-MAPPA recall rates. Breaking down the post-MAPPA recall rates, the 1-year recall to custody rate was 0.60%, the 3-year recall to custody rate was 1.87% and the 5-year recall to custody rate was 2.74%. These recall to custody figures are not high enough to account for the low proven reoffending rates reported previously, which suggests that while individuals managed under MAPPA had a higher recall rate, it is also in general more efficacious at reducing reoffending. The higher recall rates also suggest that MAPPA was effective by reincarcerating those who posed the most risk.

### Crime harm

**Figure 8:**
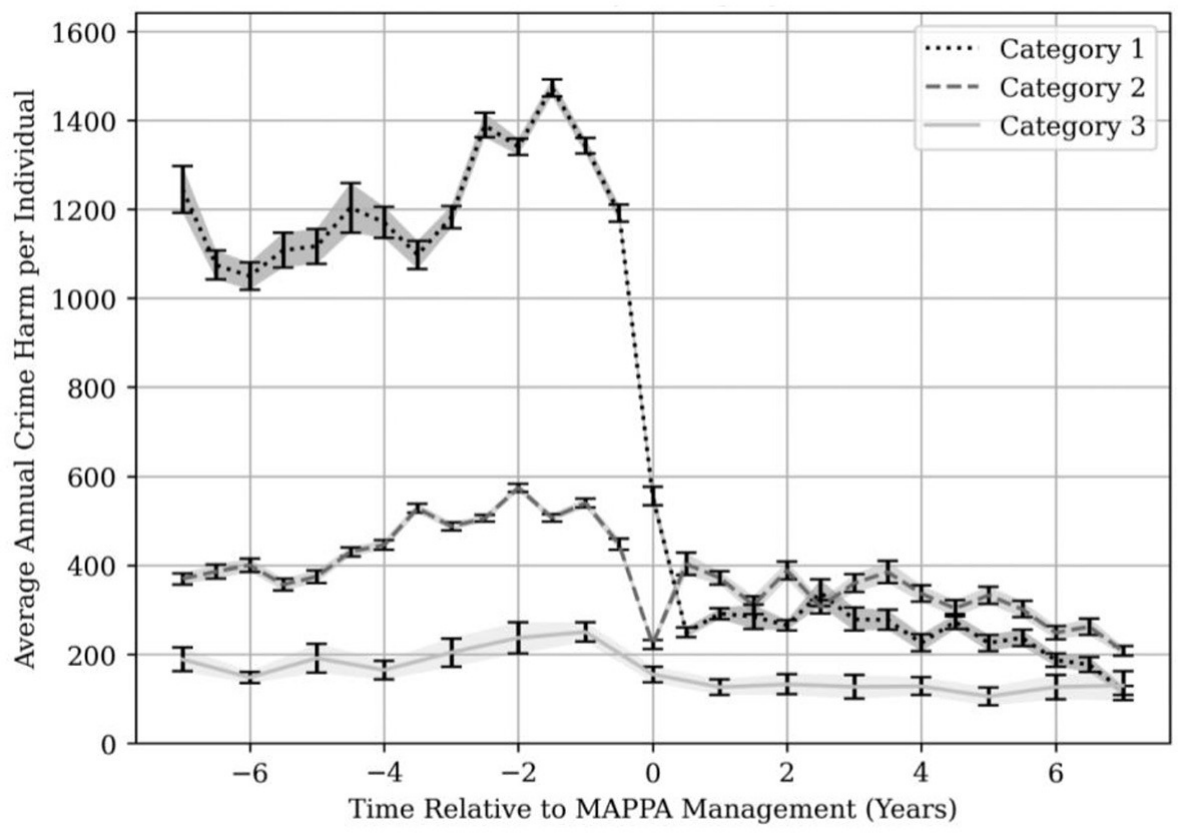
Average crime harm committed per individual per year by MAPPA category. The lines show the average annual crime harm per individual over time relative to their MAPPA registration date. For example, zero is the date of MAPPA registration,−2 is 2 years before, and +2 is 2 years post-registration. This excludes the offense of taking, making, or distributing indecent images of children.

The Cambridge Crime Harm analysis revealed that the peak average crime harm for Category 1 individuals occurs around 1.5 years before the date of registration under MAPPA and drops rapidly from this point. The large discrepancy between Category 1 and Category 2 and 3 individuals is a result of the high crime harm of many sexual offenses. In particular, the production of indecent images of children has a crime harm of 547.5 and occurs over 150,000 times in the database. This is the most commonly occurring offense; by contrast, theft from a shop, the second most common offense, occurs just under 140,000 times and has a crime harm of only 1. We therefore treated this single offense as an outlier which reduced the peak of the crime harm trend of all Category 1 offenders by over 1,000 points (a reduction of more than 40%). Figure 8 shows the results of the Cambridge Crime Harm analysis with the exclusion of the outlier offense of taking, making, or production of indecent images of children.

**Figure 9:**
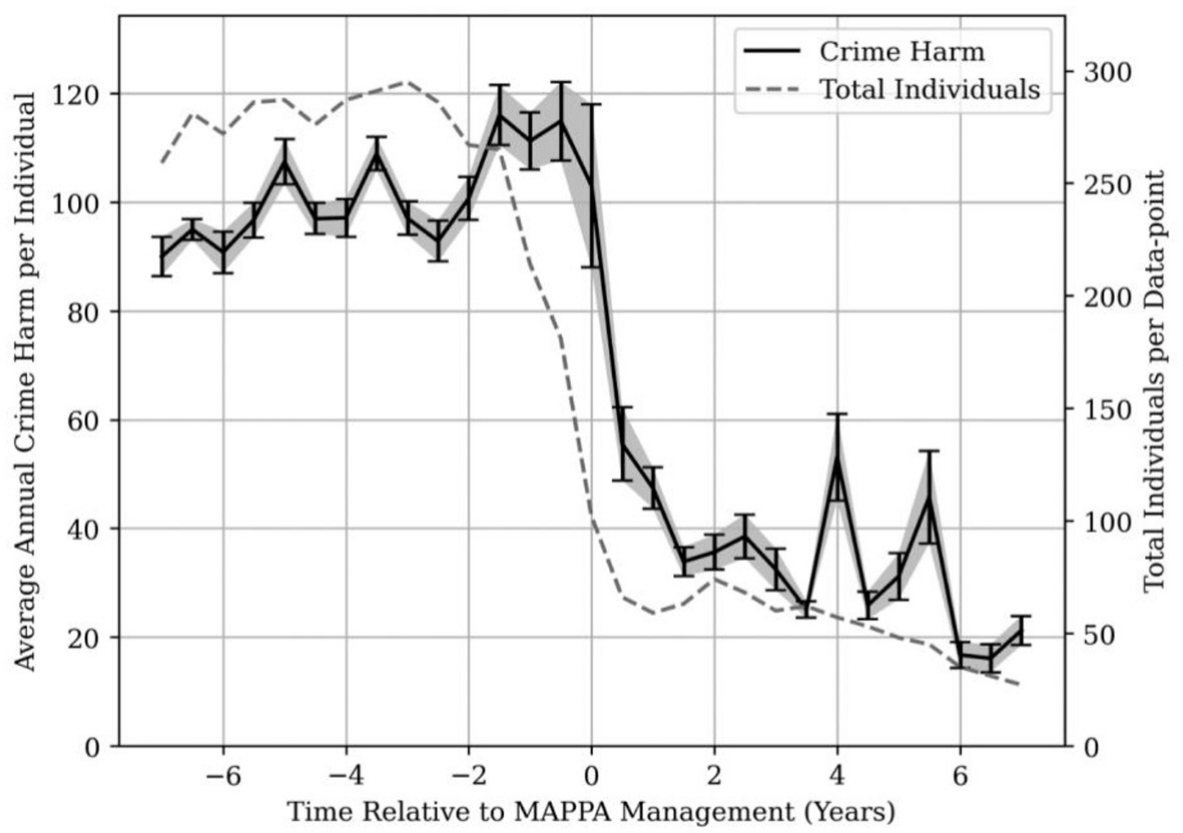
Crime harm trend for common, less serious offenses. Average crime harm trend for offenses that occur at least 20 times pre-MAPPA management, over a period of at least 3 years and with a crime harm of <30, as well as the trend in number of nominals contributing to each data point.

From Figure 8, the cause of the falling crime harm is difficult to attribute to MAPPA alone, as the downward trend could be attributed to intervention in the months leading up to MAPPA management, or alternatively put, some crimes serious enough to result in swift police intervention typically result in MAPPA management within a year or two afterwards. If we instead examine the crime harm trend not for the sample as a whole (which by extent is influenced disproportionately by frequent, high harm offenses), but rather by looking solely at low harm offenses that occur at a reliable and regular cadence over a prolonged period of time before MAPPA management, we can use such offenses to probe for phenomena (such as the advent of MAPPA management) that are likely to cause a meaningful change in their occurrence rate. By looking at Figure 9, we can see what this subset looks like as a trend in crime harm, over all MAPPA individuals.

The offset in time between the dropping number of MAPPA individuals and the dropping crime harm trend is a salient point, as it suggests that although many individuals may be suppressed in committing further crimes of all types, the remaining individuals continue to commit offenses from the selected subset routinely, with a significant drop only occurring *after* registration under MAPPA.

To verify whether the gap between the peak crime harm (for all offenses) and MAPPA management could realistically have been the result of a single serious offense committed within that timeframe, the distribution of time relative to MAPPA management for all offenses with a crime harm >500 was evaluated. It was found that 50% of such crimes were committed 3 years or fewer before MAPPA management. This drops down somewhat to 40% for 2 years or fewer, suggesting that a large fraction of such offenses are indeed being committed within the gap shown in Figure 9.

## Discussion

The aim of this research was to examine proven reoffending patterns for MAPPA-managed individuals in order to evaluate its effectiveness in reducing reoffending. The current study builds on previous research in a number of ways. First, drawing on multiple administrative data sources allowed us to access as complete a sample of MAPPA-managed individuals as possible and thus derive the most accurate proven reoffending information. This sets our research apart from previous evaluations of reoffending rates under MAPPA which drew on limited datasets and only assumed individuals' MAPPA status based on their index offense (e.g., Peck, 2011; Bryant et al., 2015). Second, and in contrast to prior research (e.g., Wood, 2012), we included MAPPA-managed individuals from all MAPPA categories available at the time of data retrieval. This included individuals in Category 2 who are supervised by Probation but are not always recorded on ViSOR. Third, our research drew on the most recent MAPPA and reoffending data from 2014 to 2021, which ensures that the reoffending rates reported within this study are reflective of the effect of the most up-to-date MAPPA guidance, especially when compared to other, large-scale evaluations of MAPPA (Peck, 2011). Fourth, we examined proven reoffending rates over a 5-year period, in contrast to previous research which examined reoffending over one – (Wood, 2012; Bryant et al., 2015) or 2-year periods (Peck, 2011). This enabled us to examine if management under MAPPA has a longer-term effect on recidivism. Fifth, we were able to examine pre- and post-MAPPA crime harm levels and recalls to custody for the same individuals. This expands upon some evaluations of MAPPA which compared reconviction rates among those individuals who had been MAPPA-assumed with those who were likely not managed under MAPPA (Peck, 2011). Lastly, and perhaps most importantly, by investigating recidivism in terms of crime harm, our study extends upon previous literature which usually measures reoffending as a dichotomous variable.

### Findings and implications

#### Effect of MAPPA on reoffending

One of the primary insights gained from our research pertains to the effects MAPPA management may have on recidivism. First, our analysis of the timing and rates of proven reoffending showed that, except for reoffending frequency, proven reoffending rates for individuals managed under MAPPA are significantly lower than those reported in overall proven reoffending statistics for England and Wales. Given that the reoffending rates are significantly lower than other publications have reported, but our average offenses committed per year by reoffenders is comparable (or higher), suggests that MAPPA is effective at preventing reoffences, but for those individuals serious enough to reoffend, there is limited effect. The finding that average reoffence numbers are higher may reflect the “type” of offender who is supervised under MAPPA, that is, it may take a particularly hardened offender for their behavior to not be affected by MAPPA management, and hence the more serious numbers in the subset that reoffend. This suggests that the right individuals are being managed under MAPPA; those who pose the greatest risk of harm.

Second, we found significant differences in pre- and post-MAPPA managed offending when utilizing a pseudo control sample made up of high frequency, low crime harm offenses. Although without a control group, it is difficult to firmly attribute this to MAPPA, it provides the best evidence thus far that management under MAPPA has a real effect on less serious offenses which do not typically involve the perpetrator's immediate removal from society. This finding also illustrates the importance of examining the role of crime harm when investigating recidivism, rather than simply measuring reoffending as a binary variable. Given that the positive effect MAPPA management may have on reducing recidivism in this population, became apparent when differences in crime harm where considered, future recidivism research may wish to routinely incorporate a measure of crime harm to establish whether such differences are also present in other contexts.

#### Individual differences in recidivism

In addition to the insights gained from examining the effects MAPPA management has on reoffending, the results of our study also provide important information regarding potential individual differences in recidivism for different “groups” of those who had offended.

Gender: First, we found that there were differences between genders in that proven reoffending rates were significantly higher for males than females. This difference in recidivism rates is perhaps unsurprising given that women account for only a small proportion (3.69%) of those adopted under MAPPA to begin with. Our findings that females are significantly less likely to reoffend align closely with the results of previous research. A well-established line of work has repeatedly demonstrated that women are less likely than men to reoffend generally (Maden et al., 2006; Freeman and Sandler, 2008; Huebner and Pleggenkuhle, 2015; Richner et al., 2023), violently (for meta-analyses, see Cortoni et al., 2010; Piquero et al., 2015; see also McCarroll et al., 2000; Ménard et al., 2009), and sexually (for a meta-analysis, see Cortoni et al., 2010; see also Sandler and Freeman, 2009). Nevertheless, there has yet to be a clear consensus on an underlying mechanism or potential mediators for this gender difference. Here, it has specifically been debated whether risk factors for reoffending are gender-neutral or gender-specific. Those endorsing a gender-neutral approach argue that the same risk factors and domains predict recidivism for males and females (for a meta-analysis, see Scott and Brown, 2018; see also Rettinger and Andrews, 2010), whilst those supporting a gender-specific approach claim that there is evidence for gender differences between predictors of recidivism (for a meta-analysis, see Collins, 2010; see also Benda, 2005; Du et al., 2013; Conrad et al., 2014; Jara et al., 2016; Miller et al., 2019; Robertson et al., 2019; Comartin et al., 2021; McNeeley, 2021; Narvey et al., 2023). Given how salient gender differences in recidivism are, as demonstrated both by previous research and our findings, it is vital that the underlying mechanisms and the differing impact of certain risk factors are investigated further. This is especially the case as there is evidence to suggest that a potential gender difference in the effect of risk factors may contribute to the fact that actuarial risk assessment tools, which were designed for use with a male population, may not be suitable for use with women who have offended (e.g., the Static-99R; Marshall et al., 2021).

Age: Second, we observed that there were significant differences in reoffending between age groups. Here, those aged 17 and under at the time of adoption under MAPPA had the significantly highest proven recidivism rates at each follow-up point. That is, reoffending rates lowered with higher age of first adoption under MAPPA. This finding is in line with Sampson and Laub's (1993) Age-Graded Theory of Informal Social Control, which posits that individuals' risk of offending varies over the life course, primarily due to informal social controls. In adulthood, individuals are required to invest social capital in relationships, the obligations of which are incongruent with continuing offending (Sweeten et al., 2010). The relationship between age and recidivism has been supported by a substantial body of evidence (for a meta-analysis, see Piquero et al., 2015). Here, higher age has been consistently linked to lower levels of general recidivism (Craig, 2011; Olver and Wong, 2015; Ambroziak et al., 2021; Van Hall, 2023), as well as violent (Richner et al., 2023) and sexual recidivism (Barbaree et al., 2003, 2009; Thornton, 2006; Nicholaichuk et al., 2014). This effect remained even when considering confounds, such as individuals' diagnoses of psychopathy (Olver and Wong, 2015), sexual arousal to offense-related stimuli (Barbaree et al., 2003), and when using brain-age measures, rather than chronological age (Kiehl et al., 2018). As a result, age has been emphasized in actuarial risk assessment scales, such as Static-99R and the Static-2002R (Helmus et al., 2012). Nevertheless, some risk assessment tools still overestimate recidivism risk for older individuals, whilst underestimating risk for younger individuals (Lussier and Healey, 2009; Wollert et al., 2010; Monahan et al., 2017). The findings of the current study suggest that all risk assessment scales need to be properly age-stratified to fully reflect the importance of age in predicting recidivism.

### Limitations

Despite the significance of our findings and their important implications for research and practice, this study had some limitations. First, whilst our aim was to capture the most comprehensive sample of MAPPA managed individuals possible across the given time-period, several issues arose that limited this. We were unable to obtain Probation Service data for Category 2 individuals who were new to MAPPA in 2012 and 2013 due to differences in how these individuals were recorded on n-Delius during this time. Therefore, it is likely that the sample was missing some of this cohort. Second, of those included in the final sample, there was also varying levels of missing data across key demographic and other characteristics. For example, ~18% of individual's records were missing ethnicity data; 70% were missing marital status information and 85% were missing data about educational attainment. Thirdly, we were not able to include a matched comparison group in our analysis and are therefore limited in the extent to which any change can be attributed to MAPPA alone.

### Future research

Our research and its findings highlighted some avenues future research may consider. First, as noted previously, the findings of this study suggest that there is a relationship between age and reoffending, which has also been well-documented in previous research. However, limited research has investigated the mechanisms and interacting variables underlying this (Doren, 2006). So far, some evidence suggests that this link between recidivism and age may differ between offense types (Hanson, 2002), and could be mediated by cognitive (e.g., proactive criminal thinking; Walters, 2022), as well as situational factors [e.g., treatment of adolescents as adults by the criminal justice system (Fowler and Kurlychek, 2018)]. In line with the Age-Graded Theory of Informal Social Control, age-related protective factors may additionally apply more to older individuals (Ambroziak et al., 2021). For instance, prosocial relationships may be more strongly related to reduced recidivism in older individuals (Lloyd et al., 2020). Given the importance of age as demonstrated by the current study, future research should investigate how risk factors for reoffending change over the life course so that age-specific criminogenic needs may be addressed (Spruit et al., 2017).

Second, we used a pseudo-control sample made up of high frequency, low crime harm offenses which showed that there were significant differences in pre-and post-MAPPA managed offending. However, without including a matched control or comparison group it is difficult to firmly attribute this effect to MAPPA alone. To address this, we are currently exploring the feasibility of further research including a comparison group drawn from individuals who were just outside of MAPPA eligibility criteria.

Third, the current study gives important insight into the contribution of MAPPA in managing individuals convicted of different types of offending. However, it was outside the scope of this project to consider views those managed under MAPPA and practitioners working with them hold toward MAPPA and its role in reducing reoffending. While previous qualitative research has examined practitioner perceptions of the effectiveness of MAPPA (Mann and Lundrigan, 2023), future qualitative research may additionally wish to investigate the viewpoint of individuals managed under MAPPA to provide further context and nuance to the existing findings.

## Conclusion

The results of this study demonstrate proven reoffending for individuals managed under MAPPA that are substantially lower than those reported in proven reoffending statistics for England and Wales. The reductions in frequency and seriousness of offending post-MAPPA management suggest that MAPPA is making a positive contribution to managing individuals convicted of sexual and violent offenses. Although without a control group, it is difficult to firmly attribute any observed change to MAPPA, this research provides the best evidence thus far that MAPPA management has a real effect on reducing less serious offenses which do not typically involve the perpetrators immediate removal from society. Future research should investigate these promising findings using a matched control design in order to determine the extent to which the findings can be attributed to MAPPA management.

## Data availability statement

The data analyzed in this study is subject to the following licenses/restrictions: The data were obtained through a pre-existing Data Sharing Agreement between Anglia Ruskin University and the National Police Chief's Council (June 2021) and are not publicly available. As such, the dataset may not be included in any submission. Requests to access these datasets should be directed to the National Police Chief's Council, https://www.npcc.police.uk.

## Ethics statement

The studies involving humans were approved by the Anglia Ruskin University's Faculty Research Ethics Panel and HMPPS' Research Ethics Committee (2020-102). The studies were conducted in accordance with the local legislation and institutional requirements. Written informed consent for participation was not required from the participants or the participants' legal guardians/next of kin in accordance with the national legislation and institutional requirements.

## Author contributions

SL: Conceptualization, Data curation, Funding acquisition, Investigation, Methodology, Project administration, Resources, Supervision, Writing – original draft. NM: Conceptualization, Data curation, Methodology, Project administration, Supervision, Writing – review & editing. DS: Data curation, Formal analysis, Investigation, Methodology, Writing – review & editing. LK: Writing – review & editing.

## Footnotes

^1^These duty-to-cooperate agencies include youth offending teams, health and social care, housing, victim support and the Home Office.

^2^In April 2022 a fourth MAPPA category was introduced for terrorist or terrorist risk individuals.

^3^ViSOR is a secure national database that was developed to support MAPPA by assisting cooperative working between the three Agencies that make up the Responsible Authority.

^4^The Police National Computer (PNC) is the computer system for England and Wales governed by section 27(4) of the Police and Criminal Evidence Act 1984. It is used to record convictions, cautions, reprimands, and warnings for any offence punishable by imprisonment and any other offence that is specified within the regulations.

^5^nDelius is a national case management information system across the Probation Service in England and Wales.

^6^The ViSOR team provided data from the start of MAPPA in 2003.

^7^The sample was missing 396 MAPPA managed individuals who do not feature on any database due their status as “restricted handling nominals.” These are individuals of public interest who have committed MAPPA eligible offences but for whom anonymity is vital.

^8^This step was only possible for the 2014 MAPPA cohort due to differences in the recording systems of Probation prior to 2014.

^9^The associated error on the reoffending value was calculated by subdividing the offender subset into ten randomly distributed bins and then taking the standard deviation of the reoffending values across each bin. A correction factor of $1/\sqrt{10}\approx 0.316$ was also introduced to the error value to account for the larger proportional error on the bins with only one tenth of the full sample size (by Poisson statistics).

^10^Individuals who are aged 18 and over and released into the community following imprisonment and some who serve a non-custodial sentence, are supervised by the Probation Service, subject to a license with conditions. If an individual fails to comply or breaches these conditions, they may be recalled back to custody.

^11^Ministry of Justice annual proven reoffending statistics are recorded from April to March whereas the present annual statistics are calculated from January to December.
